# Physiological levels of poly(ADP-ribose) during the cell cycle regulate HeLa cell proliferation

**DOI:** 10.1016/j.yexcr.2022.113163

**Published:** 2022-04-18

**Authors:** Sachiko Yamashita, Masakazu Tanaka, Chieri Ida, Kenichi Kouyama, Setsu Nakae, Taisuke Matsuki, Masataka Tsuda, Tsuyoshi Shirai, Kazuo Kamemura, Yoshisuke Nishi, Joel Moss, Masanao Miwa

**Affiliations:** a Faculty of Bioscience, Nagahama Institute of Bio-Science and Technology, Nagahama, Shiga, 526-0829, Japan; b Joint Research Center for Human Retrovirus Infection, Kagoshima University, Sakuragaoka 8-35-1, Kagoshima, 890-8544, Japan; c Department of Applied Life Sciences, College of Nagoya Women’s University, Nagoya-shi, Aichi, 467-8610, Japan; d Program of Mathematical and Life Sciences, Graduate School of Integrated Sciences for Life, Hiroshima University, Higashi-Hiroshima, 739-8526, Japan; e Pulmonary Branch, National Heart, Lung, and Blood Institute, National Institutes of Health, Bethesda, MD, 20892-1590, USA

**Keywords:** ADP-Ribosylation, Cell cycle, Cell proliferation, NAD^+^, PolyADP-ribosylation, S phase

## Abstract

Protein targets of polyADP-ribosylation undergo covalent modification with high-molecular-weight, branched poly(ADP-ribose) (PAR) of lengths up to 200 or more ADP-ribose residues derived from NAD^+^. PAR polymerase 1 (PARP1) is the most abundant and well-characterized enzyme involved in PAR biosynthesis. Extensive studies have been carried out to determine how polyADP-ribosylation (PARylation) regulates cell proliferation during cell cycle, with conflicting conclusions. Since significant activation of PARP1 occurs during cell lysis *in vitro*, we changed the standard method for cell lysis, and using our sensitive ELISA system, quantified without addition of a PAR glycohydrolase inhibitor and clarified that the PAR level is significantly higher in S phase than that in G1. Under normal condition in the absence of exogenous DNA-damaging agent, PAR turns over with a half-life of <40 s; consistent with significant decrease of NAD^+^ levels in S phase, which is rescued by PARP inhibitors, in line with the observed rapid turnover of PAR. PARP inhibitors delayed cell cycle in S phase and decreased cell proliferation. Our results underscore the importance of a suitable assay system to measure rapid PAR chain dynamics in living cells and aid our understanding of the function of PARylation during the cell cycle.

## 1. Introduction

Post-translational polyADP-ribosylation (PARylation) of proteins is initiated by members of the poly(ADP-ribose) (PAR) polymerase family (PARPs) [1] or by monoADP-ribosyl transferases [2] using NAD^+^ as the substrate. PARylation is countered by PAR glycohydrolase (PARG) and ADP-ribosyl hydrolase 3 [3–7]. PARP1, the most abundant and well-studied PARP member [1], becomes activated by exogenously added DNA-damaging agents, resulting in abundant synthesis of PAR [8–10]. In addition, inhibitors of PARylation suppress rejoining of DNA breaks that are caused by DNA-damaging agents, indicating that PARylation is involved in DNA repair [11,12]. PARP inhibitors were recently exploited clinically for the treatment of cancers, because they cause synthetic lethality in cancer cells having a genetic defect in homologous recombination [13–15]. However, resistance to PARP inhibitors is becoming another important issue [16] and the suitable application of PARG inhibitors is being proposed to circumvent the resistance to PARP inhibitors [17–19]. On the other hand, activation of PARP1 by polyanions in the presence of histone [20], or in the presence of DNA without strand breaks [21] or in the absence of DNA [22,23] has also been reported. Thus, fundamental to our understanding of PARylation, it is important to clarify the *in vivo* level of PAR, which is regulated by *in vivo* activity of PAR-synthesizing enzymes and PAR-degrading enzymes, in order to understand the relationship between the metabolism of PAR and cell proliferation under non-stressed conditions.

Previous studies indicated that cell proliferation was reduced in fibroblasts from *Parp1* knockout mice [24], and PARP inhibitors block the cell cycle at S phase [25,26]. Therefore, if PARylation is required for normal cell-cycle progression, we hypothesized that cellular PAR level is high during S phase. However, although PARP activity throughout the cell cycle has been the subject of many studies, those results are conflicting. For example, using isolated nuclei, Smulson et al. reported that PARP activity is low in S phase, increases during M phase, and peaks during G1 [27]. Other studies, however, reported that PARP activity peaks during G2 [28,29] or M phase [30]. Using nucleotide-permeabilized cells to avoid DNA breaks that can occur during the routine isolation of nuclei *in vitro* [31], Berger et al. reported that PAR biosynthesis is lowest during S phase, increases during G2/M, and peaks during G1 [32]. Of note, those previous works including ours [28] were consistent in that the PARP activity *in vitro* was relatively low in S phase. However, Hanzlikova et al. recently reported that, by enhancing immunocytochemical PAR signal using PARG inhibitor, the PAR signal was specifically observed in S phase nuclei [33]. These discrepancies may be a consequence of methodological differences and a lack of quantitative data for PAR level *in vivo*. To help address the discrepancies, we recently developed a sensitive and specific ELISA system [34,35], revealing that the levels of PAR are quite sensitive to cell lysis condition, which may cause the levels to increase by up to two orders of magnitude [34]. Thus, for quantification of the physiological level of PAR *in vivo*, it is essential that DNA damage be avoided during sample preparation and that a sensitive method to quantify PAR level be used [36].

In this study, without using PARG inhibitor, we found that the physiological concentration of PAR *in vivo* peaks during S phase and is lowest in G1. We also found that in the absence of DNA-damaging agent, PAR turns over with a half-life of <40 s, consistent with significant consumption of NAD^+^ in S phase. Our data suggest that endogenous PAR level regulates S phase progression in HeLa cells.

## 2. Materials and Methods

### 2.1. Cells and reagents

HeLa cells were provided by Riken (Tsukuba, Japan) and cultured in Dulbecco’s Modified Eagle Medium (DMEM) supplemented with 10% heat-inactivated fetal bovine serum, penicillin (100 U/ml), and streptomycin (100 μg/ml), in a humidified incubator with 5% CO_2_ at 37 °C. 3-Aminobenzamide (3AB) was obtained from Tokyo Chemical Industry (Tokyo, Japan). Olaparib was purchased from ChemScene (NJ, USA), emetine hydrochloride from Cayman Chemical (No. 21048; MI, USA), and PDD00017273 [37] from Tocris Bioscience (MN, USA), and PD0325901 from ChemScene. The following antibodies were obtained from Santa Cruz Biotechnology (TX, USA): mouse anti-human PARP1 IgG (sc-8007), mouse anti-human phosphorylated ERK IgG (sc-7383), mouse anti-human ERK2 IgG (sc-1647), horseradish peroxidase (HRP)-conjugated goat anti-mouse IgG (sc-2005), and HRP-conjugated goat anti-rabbit IgG (sc-2004). Rabbit anti-human PARG IgG (ab16060) was purchased from Abcam (Cambridge, UK). Anti-PAR (10H, IgG3 kappa), secreted from hybridoma cells [38], was purified using a Protein A Sepharose Fast Flow column (GE Healthcare, IL, USA). Anti-PAR polyclonal antibodies were produced in rabbits by injecting PAR mixed with methylated BSA in our laboratory [39,40]; the antibodies were purified with a Protein A Sepharose Fast Flow column.

### 2.2. Cell proliferation and cell viability assays

A trypan blue exclusion assay was used for quantifying cell proliferation and viability. HeLa cells (5.0 × 10^4^) were plated into 35-mm cell-culture dishes. After one day of incubation, cells were treated without or with 3AB (1–10 mM) or olaparib (1–10 μM) and further cultured for 72 h. For the cell viability assay, floating cells were combined with adherent cells that had been trypsinized, resuspended in medium, and mixed with 0.4% trypan blue solution. Viable cells were those not stained with trypan blue, as assessed by light microscopy.

### 2.3. Flow cytometry

Adherent-culture cells that had been trypsinized were centrifuged at 100×*g* for 3 min and resuspended in PBS. The suspension was fixed with ice-cold 100% ethanol to a final concentration of 70%. The suspension was centrifuged at 100×*g* for 3 min and resuspended in PBS containing 100 μg/ml RNase A, followed by staining with propidium iodide (Sigma-Aldrich). Flow cytometry was performed using a Sony Cell Sorter SH800.

### 2.4. Western blotting

Cells were washed with PBS and then fixed immediately by addition of ice-cold 20% TCA, followed by incubation on ice for 20 min. The fixed cells were collected by scraping with a plastic policeman and were centrifuged at 800×*g* for 20 min at 4 °C. The pellet was washed twice with diethyl ether, dissolved by addition of 2% SDS in 20 mM Tris-HCl (pH 8.0) and sonicated. After adjustment of protein concentration determined by a BCA kit (Thermo Scientific), each cell lysate was diluted with SDS-PAGE sample buffer, boiled for 3 min, separated by SDS-PAGE (10% polyacrylamide gels), and electrophoretically transferred to a polyvinylidene difluoride membrane. Each membrane was blocked with 2% (w/v) non-fat dry milk (Wako, Japan) or with 2% BSA in 50 mM Tris-buffered saline (pH 7.5) for 1 h at room temperature and then incubated with primary antibody overnight at 4 °C. After washing with Tris-buffered saline, each membrane was incubated with an appropriate secondary antibody in blocking buffer for 1 h at room temperature, and finally washed again with Tris-buffered saline. Immunopositive bands were visualized with HRP-conjugated antibodies and enhanced chemiluminescence using LAS 1000 or 3000 imaging system (Fujifilm, Tokyo, Japan). The intensity of the bands was quantified with Image J.

### 2.5. Synchronization of cells

For M-phase synchronization, HeLa cells (1.5 × 10^5^) were seeded into 100-mm cell-culture dishes. After incubation for 2 days, cells were cultured in medium containing 1 mM hydroxyurea for 16 h. The cells were washed twice with PBS and once with fresh medium and then incubated in fresh medium for 10 h. Then, the cells were cultured in medium containing 0.3 μg/ml (0.8 μM) colcemid for 2 h, washed two times with PBS and once with fresh medium, and then cultured in fresh medium to effect release from the G2/M boundary. For synchronization of HeLa cells at the G1/S boundary, 1.5 × 10^5^ cells were seeded into 100-mm cell-culture dishes and incubated for 2 days. Cells were then cultured in medium containing 2.5 mM thymidine for 24 h, washed twice with PBS and once with fresh medium, and then incubated for 8 h. The cells were then cultured in medium containing 2.5 mM thymidine for 16 h. Finally, the cells were washed twice with PBS and once with fresh medium and incubated with fresh medium to effect release from the G1/S boundary.

### 2.6. DNA synthesis assay

DNA synthesis was quantified with a cell-proliferation ELISA that utilized bromodeoxyuridine (BrdU) (colorimetric) (Cat. No. 11647229001, Roche Diagnostics GmbH). HeLa cells (5.0 × 10^3^) were plated into 96-well plates. After S-phase synchronization, cells were treated with 10 μM BrdU labeling solution for 15 min at 37 °C. BrdU incorporation was measured according to the Roche protocol.

### 2.7. Preparation of samples for ELISA

Cultured cells were washed with PBS, immediately fixed by adding ice-cold 20% TCA, kept for 10 min on ice, collected with a plastic policeman, and centrifuged at 800×*g* for 20 min at 4 °C. Each pellet was washed with diethyl ether, dissolved in 200 μl of 0.2 N NaOH, sonicated by Ultrasonic Homogenizer VP-5S (TAITEC Corp. Saitama, Japan) with a microtip for 10 cycles of 5 s ‘on’ and 5 s ‘off’ at a setting of 4, and incubated for 1 h at 37 °C. Each sample was added with 5 μl of 1 M Tris-HCl (pH 8.0) and 19 μl of 2 N HCl to make pH 7.5–8.0 and then incubated with 20 μg/ml DNase I, 20 μg/ml RNase A, and 1 U/ml nuclease P1 in the presence of 5 mM MgCl_2_ overnight at 37 °C. Finally, each sample was digested with 0.2 mg/ml proteinase K via incubation overnight at 50 °C, boiled for 5 min, and centrifuged at 9,700×*g* for 10 min at 4 °C. Each supernatant was frozen at −20 °C until ELISA.

### 2.8. Assay procedure for PAR with ELISA

Our ELISA for PAR was carried out essentially as described [34,35], with certain modifications. Microtiter plates had 96 wells, and were purchased from either Thermo Fisher Scientific (442404) or Nippon Genetics (TrueLine, TR5003). The wells were coated overnight with a monoclonal antibody against PAR (10H) [38] at 10 μg/ml in 50 μl coating buffer (15 mM Na_2_CO_3_ and 37 mM NaHCO_3_). The wells were blocked with 200 μl of 2% non-fat dry milk in 0.1% (v/v) Tween 20 in PBS (0.1% T-PBS) for 1 h at 25 °C and washed three times with 200 μl of 0.05% T-PBS. Samples or purified PAR standards were diluted with a buffer (0.1 M mannitol, 0.1 M NaCl and 0.1 M Tris-HCl, pH 8.0), and a 50-μl sample or standard solution containing 0, 2.5, 5, 10, 25, 50 or 75 pg PAR was added to each well with subsequent incubation for 2 h at 25 °C. The wells were washed three times with 200 μl of 0.05% T-PBS. A 50-μl volume of rabbit polyclonal anti-PAR IgG [39] was diluted to 10 μg/ml with 2% non-fat dry milk in 0.1% T-PBS and added to each well, with incubation for 2 h at 25 °C. Wells were washed three times with 200 μl of 0.05% T-PBS. A 50-μl volume of goat polyclonal anti-rabbit IgG conjugated with HRP was diluted to 0.4 μg/ml and added to each well, and the plates were incubated for 2 h at 25 °C. The wells were washed five times with 200 μl of 0.05% T-PBS, and then 100 μl *o*-phenylenediamine (0.5 mg/ml in 0.05% hydrogen peroxide solution) was added to each well with incubation for 30 min at 25 °C. The reaction was terminated by adding 25 μl of 4 N H_2_SO_4_. Then, absorbance was measured at 492 nm. The limit of detection for PAR was 2.5 pg. One picogram of PAR was defined as approximately 1.8 fmol of ADP-ribose residues [36]. Parallel cultures were used for cell counts, and PAR abundance was expressed per 10^6^ cells. When necessary, PAR level was also calculated in terms of per mg protein.

### 2.9. Determination of cellular NAD^+^ content

HeLa cells (3 × 10^4^) were seeded into 35-mm cell-culture dishes and cultured for synchronization via a double-thymidine block as described above. Extraction of NAD^+^ from cells and its quantification were carried out essentially as described by Jacobson and Jacobson [41], with minor modifications. Briefly, after washing cells with PBS, NAD^+^ was extracted by adding 0.45 ml ice-cold 0.5 N perchloric acid to dishes of cells, which were kept on ice. After 10 min, without scraping the cells, the extract was carefully aspirated with a micropipette, transferred to a microtube, and neutralized with 0.23 ml of 1 M KOH in 0.3 M potassium-phosphate buffer (pH 7.2). Microtubes were kept on ice for 10 min and then centrifuged at 9,700×*g* for 10 min at 4 °C. Each supernatant was stored at −20 °C until determination of NAD^+^. For that purpose, 50 μl of each thawed sample or NAD^+^ standard (0.2–1.0 μg/ml) were added into wells of a 96-well plate, with subsequent addition of 100 μl of freshly prepared assay mixture consisting of 100 mM Na-Bicine (pH 7.8), 0.5 mM MTT tetrazolium (Sigma-Aldrich), 2 mM phenazine ethosulfate (Sigma-Aldrich), 5 mM EDTA, 1 mg/ml BSA, and 500 mM ethanol. The enzymatic cycling assay was initiated by adding 10 U alcohol dehydrogenase (Cat. No. 46410005, Oriental Yeast Co., Tokyo) in 20 μl of 100 mM Na-Bicine (pH 7.8). Samples were incubated for 30 min at 37 °C, and the absorbance at 570 nm was determined with a reference at 650 nm.

### 2.10. Purification of human PARP1

Human PARP1 was produced in Rosetta (DE3) pLysS competent *Escherichia coli* expressing the recombinant plasmid containing human *PARP1* cDNA (from Dr. Michael Ranes). PARP1 was purified with a Ni Sepharose (HisTrap HP) column and a HiTrap Heparin HP column.

### 2.11. Measurement of PARP1 activity in vitro by ELISA

PARP1 activity *in vitro* was measured by formation of immunoreactive PAR. Each reaction mixture (25 μl) contained 100 mM Tris-HCl, pH 8.0, 10 mM MgCl_2_, 1 mM DTT, 100 μg/ml BSA, and 2.5 pmol of the purified human PARP1 (0.1 μM) (Fig. 8) in the presence or absence of 200 pmol human histone H4 and/or H2A (8 μM). The reaction was initiated by adding 2 mM NAD^+^ in the presence or absence of 10 μM olaparib, with incubation for 30 min at 37 °C. Then 50 μl of 10 mg/ml BSA and 500 μl of 20% TCA were added. The resultant precipitates were recovered by centrifugation at 9,700×*g* for 5 min at 4 °C, washed with 500 μl diethyl ether, dissolved in 200 μl of 0.2 N NaOH, and sonicated with a microtip for 10 cycles of 5 s ‘on’ and 5 s ‘off’ at a setting of 4. The following procedures were the same as those described above, except that the treatment with DNase I, RNase A, and nuclease P1 was omitted for experiments shown in Fig. 8. A 100-bp DNA with a nick in the middle of the sequence (Nb.BsmI site of pBR322, underlined) was prepared by annealing with 100 nt of 5′-caccactccaagaattggagccaatcaattcttgcggagaactgtgaatgcgcaaaccaacccttggcagaacatatccatcgcgtccgccatctccagc-3′, 50 nt of 5′-gctggagatggcggacgcgatggatatgttctgccaagggttggtttgcg-3′ and 50 nt of 5′-cattcacagttctccgcaagaattgattggctccaattcttggagtggtg-3’.

### 2.12. Statistical analysis

The data are presented as the mean ± standard deviation, and the statistical significance of the difference between subjects was evaluated with Student’s two-tailed *t*-test for comparing the significance between two subjects, one-way ANOVA, Dunnett’s multicomparison test, and Tukey’s multicomparison test for comparing the significance among more than two subjects. The test of no correlation (corr.test function) with the R statistical package was also used [42].

## 3. Results

### 3.1. Inhibition of cell proliferation by a nontoxic concentration of PARP inhibitors, 3AB and olaparib

**Fig. 1.**
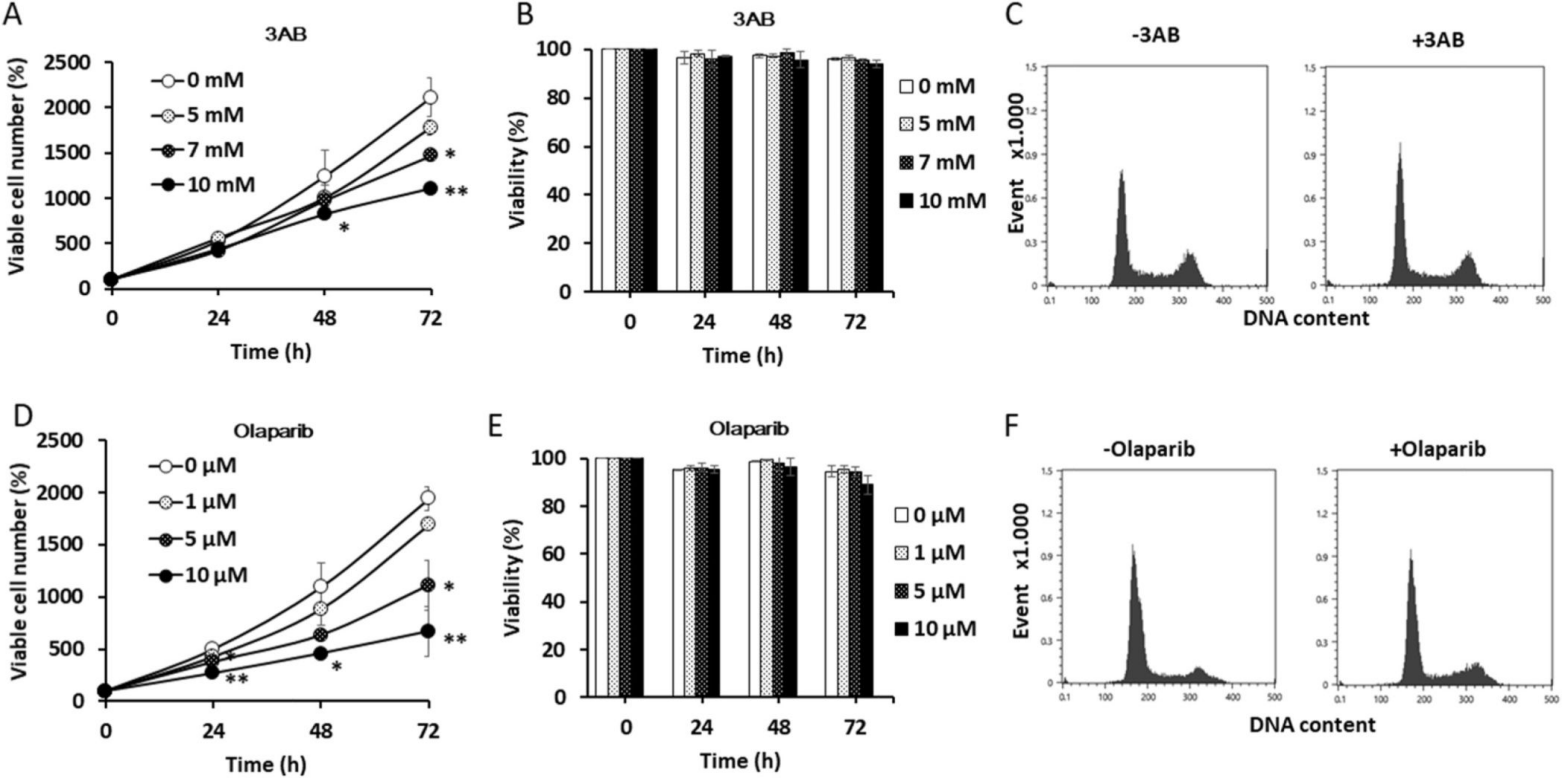
Inhibition of cell proliferation by nontoxic concentration of PARP inhibitors, 3AB and olaparib. Proliferation of HeLa cells was determined at 24, 48, and 72 h after treatment without or with 3AB (n = 3) in (A) and with olaparib (n = 2) in (D). Cells (5.0 × 10^4^ cells) were seeded into 35-mm cell-culture dishes one day prior to treatment. A specified number of viable cells, namely 5.0 × 10^4^ viable, was defined as 100% (time 0). The significance was analyzed against the control by one-way ANOVA and Dunnett’s multicomparison test. *p < 0.05, **p < 0.01. The viability of cells treated with 3AB (n = 3) or olaparib (n = 2) is shown in (B) and (E), respectively. The significance was analyzed against the control by one-way ANOVA and Dunnett’s multicomparison test. (C) and (F) Representative flow cytometry profiles for asynchronously proliferating HeLa cells treated for 24 h without or with 10 mM 3AB (n = 3) or 5 μM olaparib (n = 2); G1, 62.7 ± 2.0%; S, 15.1 ± 2.1%; G2/M, 22.3 ± 2.6% in the absence of 3AB (Fig. 1C, left panel) and G1, 58.5 ± 0.8%; S, 15.9 ± 1.9%; G2/M, 25.7 ± 1.2% (Fig. 1C, right panel) in the presence of 3AB; G1, 75.7 ± 1.4%; S, 12.8 ± 0.4%; G2/M, 11.5 ± 1.1% in the absence of olaparib (Fig. 1F, left panel) and G1, 64.9 ± 0.3%; S, 17.8 ± 0.7%; G2/M, 17.3 ± 0.5% in the presence of olaparib (Fig. 1F, right panel). The significance was analyzed against the control by two-tailed *t*-test.

We first confirmed published data pertaining to the effect of two PARP inhibitors on HeLa-cell proliferation; these inhibitors were 3AB, which alone is neither mutagenic nor cytotoxic [25,43,44], and olaparib, which was recently applied clinically [15]. Incubation of cells with 3AB at 7 or 10 mM significantly suppressed proliferation after 72 h (Fig. 1A). However, no significant effect of 3AB on cell viability was observed over the entire concentration range tested (0–10 mM; Fig. 1B). This result confirmed that 3AB was not cytotoxic. Flow-cytometry profiles for asynchronously growing HeLa cells did not differ significantly in the presence of 10 mM 3AB for 24 h (Fig. 1C). Olaparib, which specifically inhibits PARP1 and PARP2 [45] and thus is used for cancer therapy [15], also significantly suppressed cell proliferation at 5 μM and 10 μM (Fig. 1D). As with 3AB, however, olaparib did not significantly affect cell viability after 72 h (Fig. 1E). The decrease for G1 (p < 0.001), increase for S (p < 0.001), and increase for G2/M (p < 0.05) in the presence of olaparib were statistically significant. These results confirmed that a non-cytotoxic dose of either of two PARP inhibitors significantly inhibited cell-cycle progression.

### 3.2. PARP inhibitors block cell-cycle progression at S phase

The level of cyclin A correlates with the rate of DNA replication [46, 47]. Thus, to determine the cell-cycle phase that is blocked by PARP inhibitors, we measured the levels of cyclin A and cyclin B1 as indicators of cell-cycle progression through S phase and G2/M, respectively. Cyclin A signal was increased at 4 h and 8 h after release from a double-thymidine block but was quite faint at 12 h after release. However, it was still present with 3AB or olaparib at 12 h ( Fig. Supporting information Fig. S1A and B). Similar results were obtained for cyclin B1. These data suggested that PARP inhibitors block the cell cycle at S phase or G2/M or both.

**Fig. 2.**
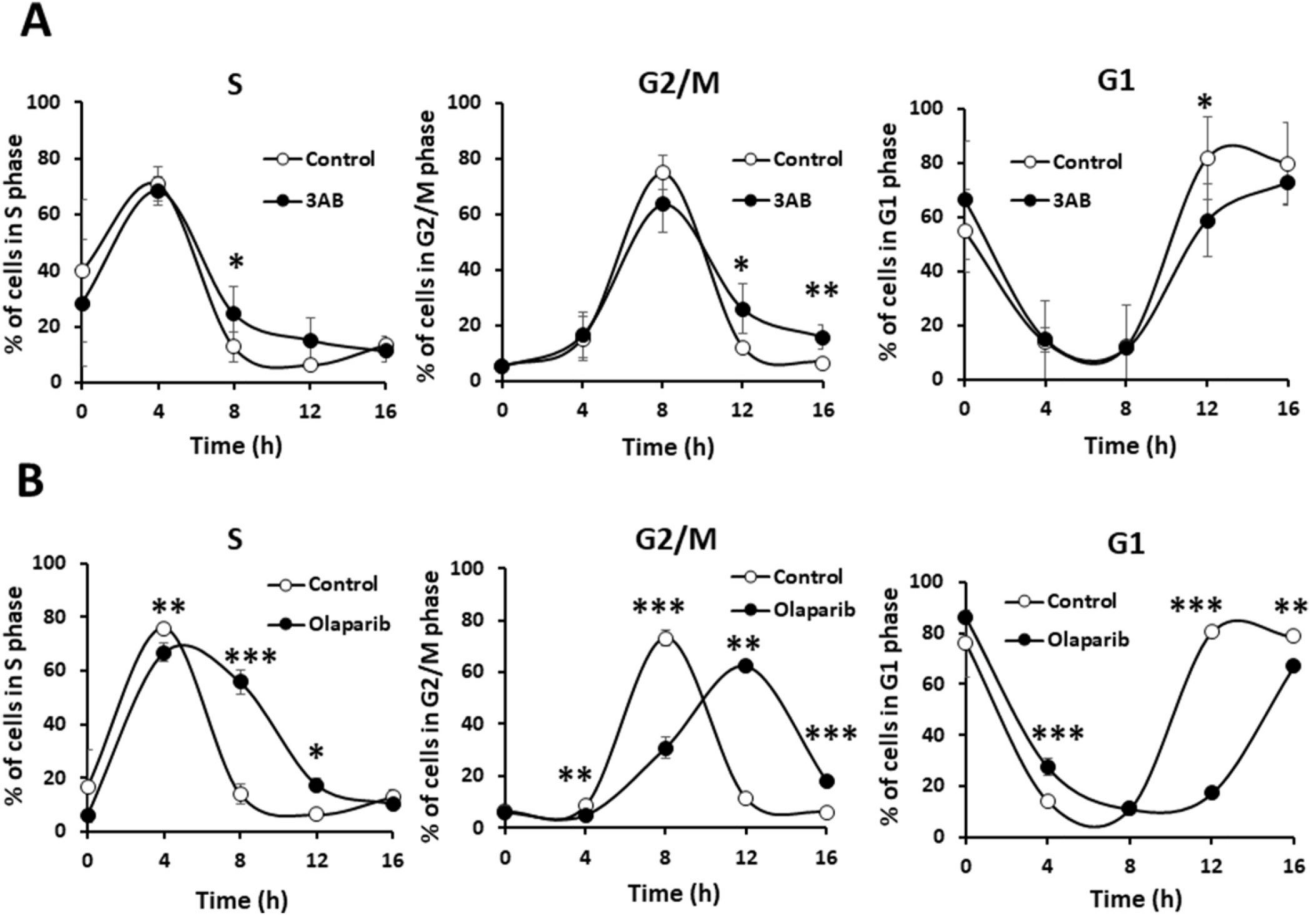
3AB and olaparib block cell-cycle progression in S phase after release from the G1/S boundary. Flow cytometric analysis at different time points after release from the G1/S boundary without or with 10 mM 3AB (n = 5) in (A) or 5 μM olaparib (n = 2) in (B). The percentage of cells in each phase is calculated from DNA histograms. The results represent the mean ± standard deviation. The significance was analyzed against the control without 3AB or olaparib by two-tailed *t*-test. *p < 0.05, **p < 0.01, ***p < 0.001.

To determine the exact cell-cycle phase that is affected by each PARP inhibitor, we analyzed the cell-cycle progression using flow cytometry after cells were treated with a nontoxic level of each inhibitor, i.e., 10 mM 3AB and 5 μM olaparib. At 4 h after release from the G1/S boundary, 3AB did not significantly affect the percentage of the S-phase population (Fig. 2A). At 8 h after release, however, the S-phase population was larger with 3AB (24.7 ± 9.7%) than without 3AB (12.8 ± 5.5%), implying a significant prolongation of S phase (p < 0.05) (Fig. 2A, left). This delay carried over to G2/M (12 h post-release) and G1 (16 h) (Fig. 2A, middle and right). The block at S phase occurred sooner with 5 μM olaparib. As early as 4 h after release, the S-phase population was 76.0 ± 1.7% without olaparib but 66.9 ± 3.3% with olaparib (p < 0.01) (Fig. 2B, left), and the difference was more pronounced after 8 h (p < 0.001) and 12 h (p < 0.05), consistent with the prolongation of S phase. This delay also carried over to G2/M and to G1 (Fig. 2B, right). These results confirmed that PARP inhibitors suppress cell-cycle progression, causing a block mainly in S phase.

### 3.3. PAR levels in the presence of a DNA-damaging agent

To confirm the importance of avoiding DNA damage during cell harvest/lysis for quantification of PAR level *in vivo* [34], we compared the effect of the DNA-damaging agent *N-*methyl-*N*′-nitro-*N*-nitrosoguanidine (MNNG) on PAR level of asynchronized HeLa cells. Nontreated cells had a low level of PAR, i.e., 60.3 ± 43.1 pg/10^6^ cells, but treatment with MNNG (0.3–10 μM) for 30 min increased the level as follows (per 10^6^ cells): 0.3 μM, 109 ± 38 pg; 1 μM, 254 ± 104 pg; 3 μM, 1,530 ± 697 pg; 10 μM, 4,020 ± 1,270 pg ( Fig. Supporting information Fig. S2). This large increase in PAR level after MNNG treatment is consistent with work in SV40-transformed 3T3 cells [48], C3H10T1/2 cells [49], and HeLa cells [34]. These results clearly demonstrated that DNA damage should be avoided when preparing cell lysates and that methods more sensitive than Western blotting are necessary for quantifying endogenous PAR (see below).

### 3.4. PAR levels vary during the cell cycle

**Fig. 3.**
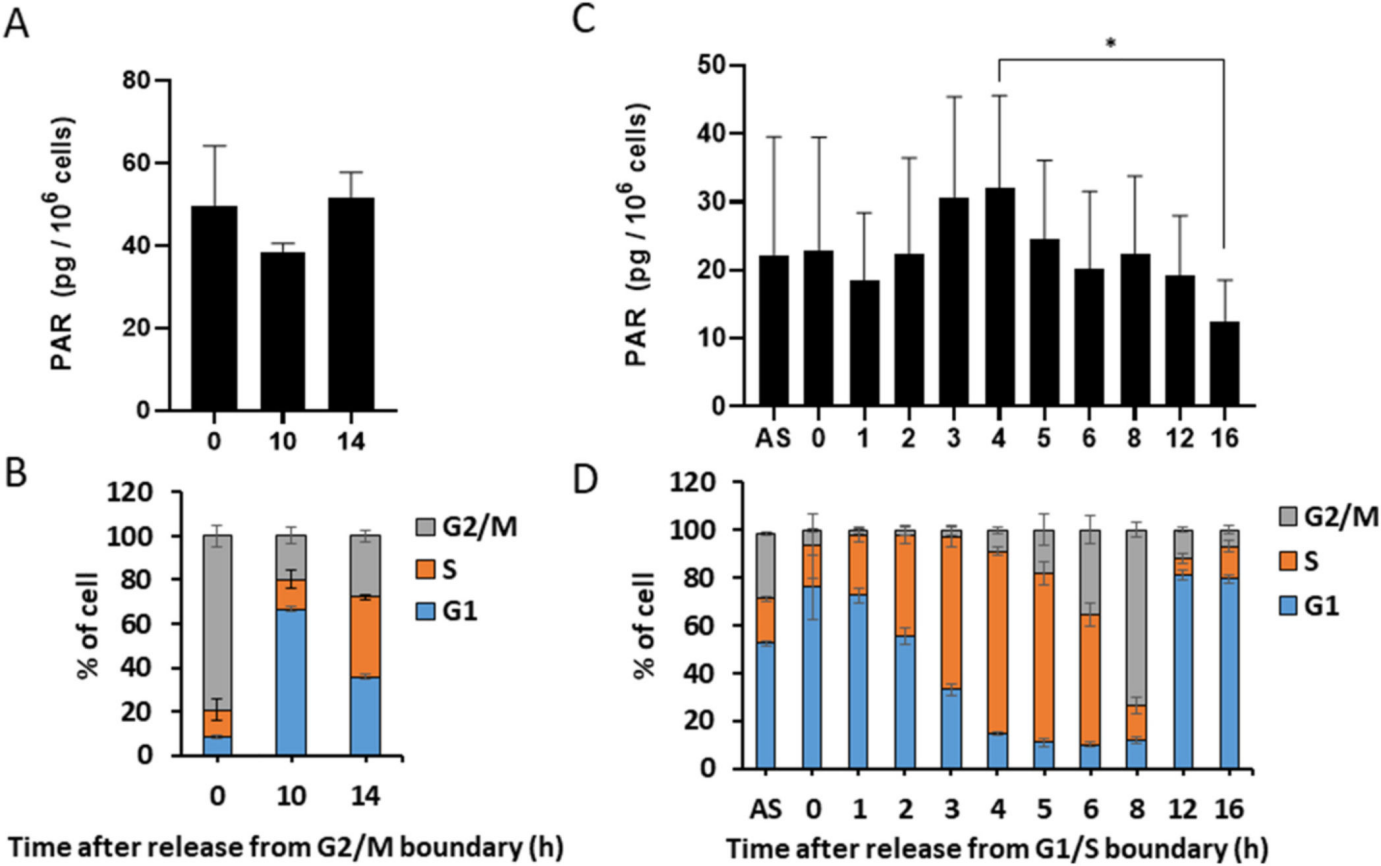
*In vivo* level of PAR in HeLa cells peaks during S phase and is lowest in G1. (A) Time course of PAR levels per 10^6^ cells after release from M phase. n = 4. (B) Histogram of cell populations after release from M phase. n = 3. (C) Time course of PAR levels per 10^6^ cells after release from the G1/S boundary. n ≥ 4. (D) Histogram of cell populations after release from the G1/S boundary. n ≥ 3. The results represent the mean ± standard deviation. The significance was analyzed by one-way ANOVA and Tukey’s multicomparison test for (A) and (C), and by the test of no correlation with the R statistical package ( Fig. Supporting information Fig. S4). *p < 0.05.

We next analyzed PAR level in proliferating HeLa cells throughout the cell cycle. Cells were synchronized at G2/M with hydroxyurea and colcemid, and PAR levels were measured over time after release from colcemid (per 10^6^ cells): Time 0, 49.8 ± 14.5 pg; 10 h, 38.5 ± 2.1 pg; 14 h, 52.0 ± 5.9 pg (Fig. 3A). The PAR level after 14 h was greater than that at 10 h, suggesting that PAR level peaks during S phase (Fig. 3B).

The PAR level was also determined in HeLa cells synchronized by the double-thymidine block method to enrich each cell-phase population (Fig. 3C and D), which yielded the following cell-cycle populations: S phase (4 h after release from the G1/S boundary), 76.4 ± 1.7%; G2/M (8 h) 73.6 ± 3.0%; G1 (12 h), 81.0 ± 2.0%. Consistent with these data, BrdU incorporation, which reflects the rate of DNA synthesis, was very low at time 0, but increased continuously until 6 h after release from the G1/S boundary ( Fig. Supporting information Fig. S3).

At 1 h after release from the G1/S boundary, the PAR level was 18.6 ± 9.9 pg/10^6^ cells, which was lower than that at time 0 (23.0 ± 16.5 pg/10^6^ cells). The level began to increase 2 h after release and peaked after 4 h (maximum S-phase population) at 32.0 ± 13.6 pg/10^6^ cells (0.051 ± 0.025 pmol/10^6^ cells) (Fig. 3C and D); this value gradually decreased until 6 h after release (20.1 ± 11.5 pg/10^6^ cells), increased slightly at 8 h (G2/M) (22.4 ± 11.4 pg/10^6^ cells), then decreased through 12 h (G1) (19.2 ± 8.9 pg/10^6^ cells) and 16 h (G1) (12.6 ± 6.0 pg/10^6^ cells). PAR level at 4 h after release (S) was significantly greater than that at 16 h (G1) analyzed by one-way ANOVA and Tukey’s multicomparison test (p <0.05) (Fig. 3C). In addition, there was a significant positive correlation between PAR level/cell and S phase cells population (r = 0.72; 95% confident interval, 0.21–0.92; p = 0.013) by the test of no correlation with the R statistical package, while there was no significant correlation between PAR level/cell and G2/M or G1 cells population ( Fig. Supporting information Fig. S4). When PAR level was expressed per mg protein of cells, the PAR level at 4 h after release (S) was 67.0 ± 26.0 pg (0.123 ± 0.048 pmol/mg protein) and 42.8 ± 12.9 pg/mg protein at 16 h (G1), and there was a significant positive correlation between PAR level/protein and S phase cells population (p = 0.012) by the test of no correlation with the R statistical package. These results demonstrated that physiological PAR level peaked during S phase and was lowest in G1.

### 3.5. PAR chain dynamics with PARP inhibitors during the cell cycle

**Fig. 4.**
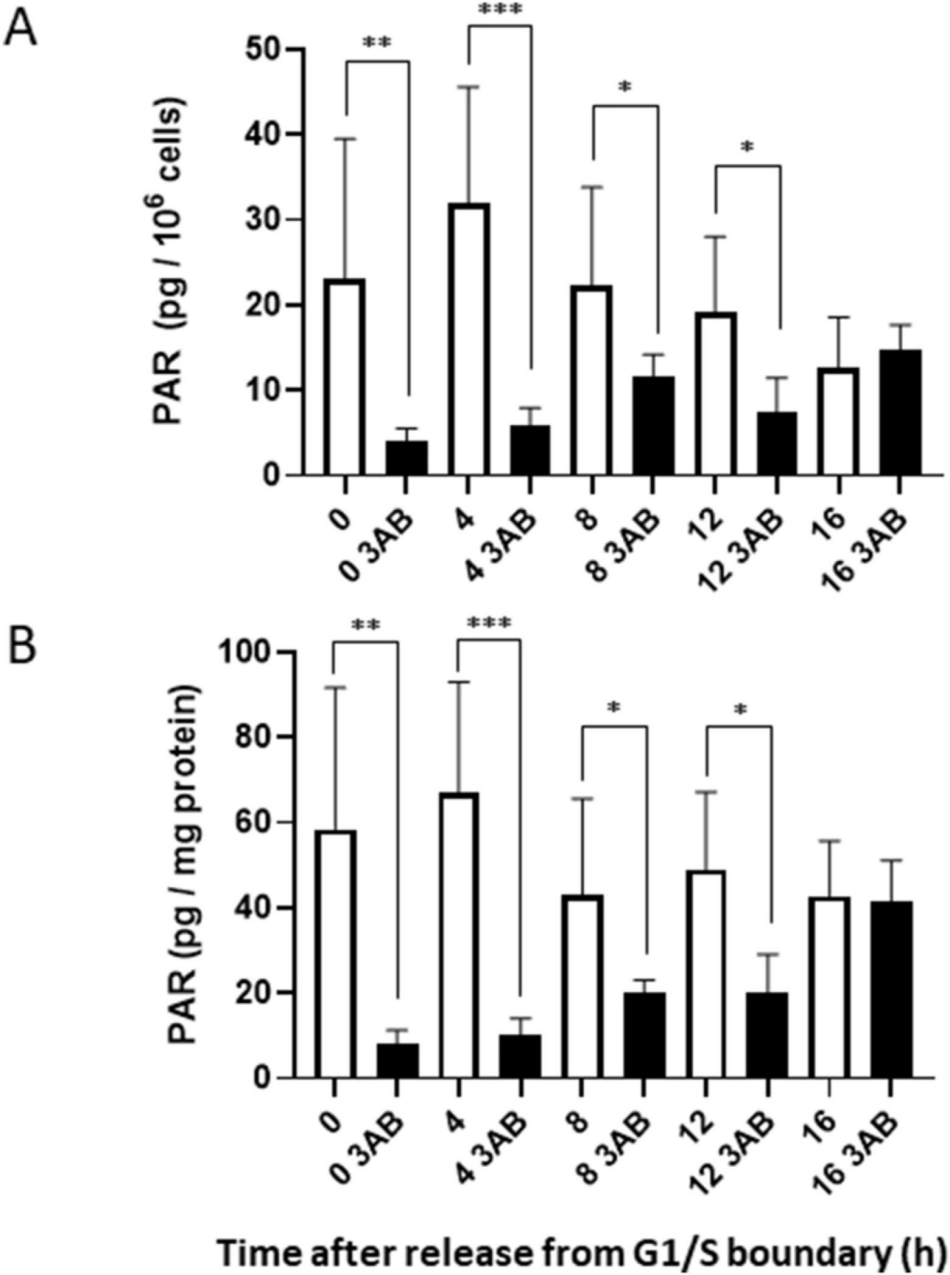
PAR levels after release from the G1/S boundary in the absence or presence of 10 mM 3AB. HeLa cells were cultured without (white column) or with (black column) 10 mM 3AB for the indicated time, washed with PBS, fixed with 20% TCA, and processed for ELISA. PAR levels are shown per 10^6^ cells in (A) and per mg protein in (B). The significance was assessed between PAR levels measured without or with 3AB by two-tailed *t*-test. n ≥ 4. *p < 0.05, **p < 0.01, ***p < 0.001.

Although the half-lives of PAR in cells treated with DNA-damaging agents were reported as less than 1 min–6 min [48,50,51], the turnover rate of PAR under unperturbed physiological conditions has not been determined. Therefore, 3AB was applied to synchronized HeLa cells to determine how PARP inhibitor affects PAR half-life and how long the inhibitor could suppress PAR level throughout the cell cycle. Intriguingly, treatment of cells with 10 mM 3AB caused the PAR level to decrease rapidly (within 40 s; 0 h) from 23.0 ± 16.5 pg/10^6^ cells to 4.0 ± 1.5 pg/10^6^ cells and from 58.5 ± 33.1 pg/mg protein to 8.3 ± 3.0 pg/mg protein (Fig. 4A and B). Considering such time for washing out the thymidine, adding new medium containing 3AB, and immediately washing with PBS before addition of TCA to stop PAR metabolism at 0 h, the actual half-life of PAR should be much shorter.

Relative PAR levels with 3AB to that without 3AB at each time, were as follows: 18% (4 h after release from the G1/S boundary), 51% (8 h), 39% (12 h), and 118% (16 h) (per 10^6^ cells) (Fig 4A); 15%, 47%, 41% and 97% (per mg protein) (Fig. 4B).

### 3.6. PARP1 and PARG levels do not change significantly during the cell cycle

To understand the mechanism by which PAR level peaks during S phase, Western blotting was used to quantify cellular PARP1, as it is the major enzyme responsible for PAR biosynthesis [52]. Relative to time 0 (band intensity = 1.0), PARP1 band intensity after release from the G1/S boundary was as follows: 4 h, 1.04 (±0.29); 8 h, 1.15 (±0.52); 12 h, 0.97 (±0.21) ( Fig. Supporting information Fig. S5); these values did not differ significantly, consistent with results reported by Yamanaka et al. as assessed in the human lymphoblastoid cell line, CEM [53]. Western blotting was also used to assess the level of PARG, which is the major enzyme involved in PAR degradation. Relative PARG band intensity after release from the G1/S boundary was as follows: 4 h, 1.09 (±0.12); 8 h, 1.13 (±0.10); 12 h, 1.10 (±0.25) ( Fig. Supporting information Fig. S5); these values did not differ significantly. Therefore, because PARP1 and PARG levels do not vary significantly throughout the cell cycle, the observed increased level of PAR in S phase must be a consequence of another factor(s).

### 3.7. NAD^+^ level decreases significantly during S phase and recovers by PARP inhibitors

**Fig. 5.**
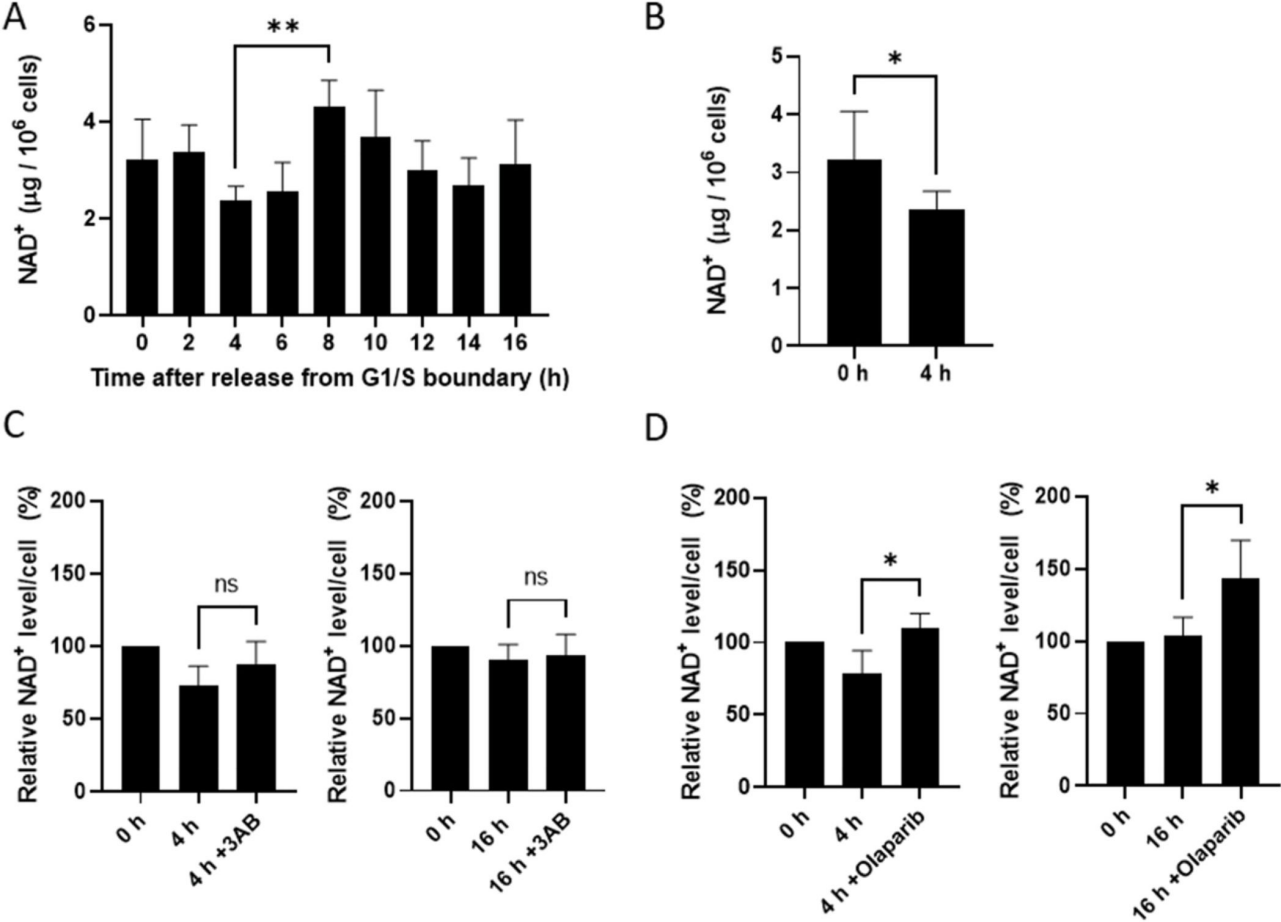
NAD^+^ level decreases during S phase of the cell cycle and PARP inhibitors prevent its decrease. HeLa cells were synchronized by a double-thymidine block. NAD^+^ was extracted at the indicated times after release from the G1/S boundary. NAD^+^ was determined by cycling assay as described in Materials and Methods and expressed as μg/10^6^ cells. (A) The significance was analyzed with ANOVA and Tukey’s multicomparison test. n ≥ 3. **p < 0.01. (B) NAD^+^ levels at 0 h and 4 h were 3.2 ± 0.7 and 2.4 ± 0.3 μg/10^6^ cells, respectively. The significance was analyzed with two-tailed *t*-test. n ≥ 7. *p < 0.05. (C) HeLa cells were added with the medium without or with 10 mM 3AB at 0 h after release from G1/S boundary and cultured for 4 h (S phase) or 16 h (G1 phase) before extraction of NAD^+^. (D) HeLa cells were added with 3.5 mM dimethyl sulfoxide (DMSO) as vehicle or with 5 μM olaparib in 3.5 mM DMSO, and cultured for 4 h (S phase) or 16 h (G1 phase) before extraction of NAD^+^. (C) and (D) NAD^+^ levels/cell were expressed as percentages using the value at 0 h as 100%. The significance was analyzed with two-tailed *t*-test. n = 4. ns, not significant. *p < 0.05.

Because PAR biosynthesis by PARPs requires NAD^+^, it is possible that NAD^+^ level increases during S phase. However, the NAD^+^ level in mid-S phase (4 h) of 2.4 ± 0.3 μg (3.6 ± 0.5 nmol)/10^6^ cells was the lowest among all cell-cycle phases (Fig. 5A). Conversely, the NAD^+^ level at 8 h (G2/M) of 4.3 ± 0.6 μg (6.5 ± 0.9 nmol)/10^6^ cells was the highest among all phases. This low level of NAD^+^ in S phase is consistent with results reported for the human amnion cell line, FL [54]. Further experiments also showed the significant difference in NAD^+^ levels between 0 h and 4 h (Fig. 5B). And PARP inhibitors, 3AB and olaparib, prevented the decrease of NAD^+^ levels in S phase (Fig. 5C and D). Walker reported an NAD^+^ level of 1,350 pmol/10^6^ cells in asynchronized KB cells, a HeLa cell line [55]. Two reports have estimated the average volume of an individual HeLa cell: 6,896 μm^3^ (6.896 × 10^−12^ l) [56], and 2,425 μm^3^ (2.425 × 10^−12^ l) [57]. Based on these calculations, the concentration of NAD^+^ during the cell cycle varies between 0.5 and 0.9 mM or between 1.5 and 2.7 mM, respectively. Considering that the Km value for human PARP1 for NAD^+^ is ~50 μM *in vitro* [58], NAD^+^ concentration can neither be rate limiting nor explain the observed increase in PAR level *in vivo* during S phase.

### 3.8. MEK inhibitor does not prevent the increase in PAR level during S phase

**Fig. 6.**
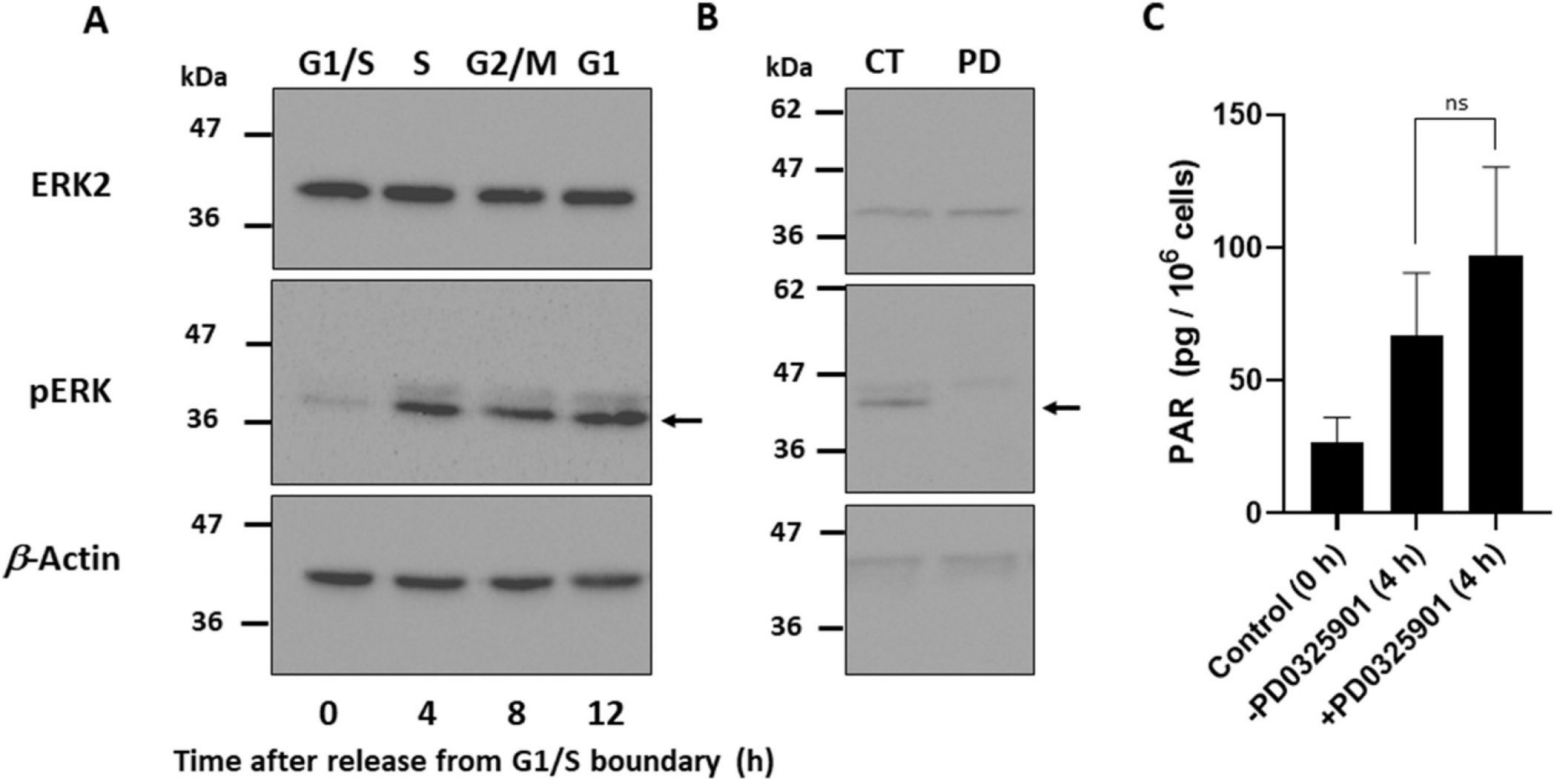
Phosphorylated ERK2 level increases at 4 h post-release, but a MEK inhibitor does not suppress the elevation in PAR level during S phase. HeLa cells were synchronized by a double-thymidine block. (A) ERK2, pERK and β-actin were detected with respective antibodies. The representative data from three independent experiments are presented. (B) Effect of a MEK inhibitor on pERK2 level. After release from the G1/S boundary, the culture medium was replaced with fresh medium containing 10 μM MEK inhibitor (PD0325901) in 0.005% ethanol (PD). Medium containing 0.005% ethanol without the inhibitor (CT) was prepared as the control. The cells were cultured for 4 h and then subjected to Western blotting using the respective antibodies. The representative data from three independent experiments are presented. The arrows in (A) and (B) indicate pERK2, and the upper bands indicate pERK1. β-Actin was used as a loading control. (C) HeLa cells were synchronized with double-thymidine block. The medium was replaced by a fresh medium containing 0.005% ethanol without or with 10 μM PD0325901, and the cells were collected at 0 h and 4 h (S phase) after release from G1/S boundary for determination of PAR levels by ELISA. The significance of changes of PAR levels 4 h after release was analyzed without and with MEK inhibitor by two-tailed *t*-test. n = 5. ns, not significant.

Because PARP1, PARG, and NAD^+^ levels could not explain the increase in PAR level during S phase, we searched the literature for factors that could potentially explain the increase in PAR level during S phase *in vivo* in the absence of DNA-damaging agents or in the absence of DNA. Cohen-Armon et al. reported that pERK2, which contributes to mitogen-activated protein kinase signaling pathway, can bind to and activate PARP1 *in vitro* in the absence of DNA and that PARP1-ERK synergism exists in proliferating cells [22,59]. The pERK2 band intensity at 4 h post-release from the G1/S boundary (S phase), was significantly greater than that at 0 h by 2.8 (±0.7)-fold (p < 0.05) (Fig. 6A). If the increase in pERK2 level was the cause of PARP1 activation during S phase *in vivo*, then the mitogen-activated protein kinase kinase (MEK) inhibitor should inhibit the increase in PAR level in S phase. The MEK inhibitor, PD0325901, at 10 μM inhibited the increase in pERK2 level during S phase, as expected (Fig. 6B). Then we asked whether PAR level decreases with treatment of the MEK inhibitor. PAR levels without PD0325901 at time 0 and 4 h post-release (S phase) were 26.5 ± 9.5 pg/10^6^ cells and 67.0 ± 23.6 pg/10^6^ cells, respectively. In the presence of PD0325901, however, PAR level at 4 h (97.3 ± 33.2 pg/10^6^ cells) did not decrease (n = 5, p = 0.13) and rather tended to increase as compared to the control level at 4 h (Fig. 6C). Thus, the observed increase in PAR during S phase *in vivo* is independent of pERK2.

### 3.9. The protease inhibitor emetine does not prevent the increase in PAR level during S phase

**Fig. 7.**
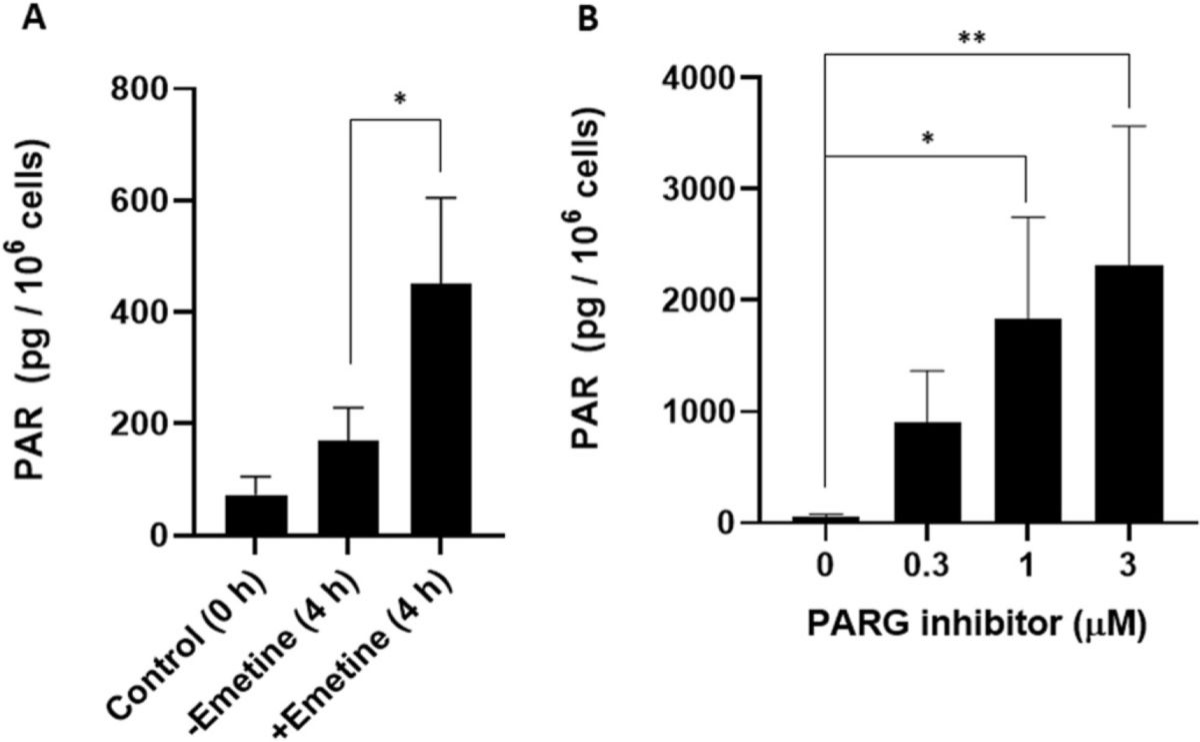
Emetine does not inhibit the increase in PAR level during S phase, and a PARG inhibitor greatly increases PAR level. (A) HeLa cells were synchronized by a double-thymidine block. The culture medium was replaced by fresh medium containing 2 μM emetine (2 mM emetine in ethanol was diluted with culture medium) at 3 h after release from the G1/S boundary, with culture for an additional 1 h. The cells were then collected 4 h after release (S phase) for ELISA. PAR levels without or with emetine from four independent experiments are shown. The significance of changes of PAR levels 4 h after release was analyzed without and with emetine using two-tailed *t*-test. *p < 0.05. (B) HeLa cells were cultured asynchronously in 100-mm dishes, and the medium was replaced with DMEM containing 10% fetal bovine serum along with the indicated concentration of the PARG inhibitor, PDD00017273, dissolved in 0.1% DMSO. The cells were cultured for 30 min at 37 °C, washed with PBS, immediately fixed by adding ice-cold 20% TCA, and processed for ELISA. The significance of the changes of PAR levels after addition of the designated concentration of PARG inhibitor was analyzed with one-way ANOVA and Dunnett’s multicomparison test. n = 4. *p < 0.05, **p < 0.01.

Because the basal PAR level is quite low, Hanzlikova et al. immunocytochemically enhanced endogenous PAR signals by inhibiting PAR degradation using the cell-permeable PARG inhibitor, PDD00017273. The results revealed that PAR signals were found in the nuclei of proliferating cell nuclear antigen (PCNA)-producing cells of human diploid RPE-1 cells, U2OS cells and HeLa cells, after pre-extracted in 0.2% Triton-X-100 before fixation, suggesting the endogenous PAR synthesis in S phase [33]. They then hypothesized that unligated Okazaki fragments at DNA replication forks could activate PARP1 and PARP2 to synthesize PAR during S phase; this was posited because emetine, a protease inhibitor that inhibits Okazaki-fragment synthesis specifically at 2 μM [60], caused disappearance of PAR signals during S phase in both RPE-1 and U2OS cells in the presence of 10 μM PDD00017273 [37]. We tested this hypothesis using our synchronized cell system. At 3 h post-release from the G1/S boundary, the culture medium was replaced with medium lacking or containing 2 μM emetine, and the incubation continued for 1 h. TCA was then added to fix the cells in S phase (i.e., 4 h after release from the G1/S boundary), and PAR level was determined by ELISA. PAR levels without emetine at time 0 h and 4 h post-release (S phase) were 72.1 ± 32.9 pg/10^6^ cells and 170 ± 58.9 pg/10^6^ cells, respectively. In the presence of emetine, however, PAR level at 4 h (450 ± 155 pg/10^6^ cells) was not decreased but significantly increased than that of the control (p < 0.05) (Fig. 7A). In addition, when asynchronously growing HeLa cells were treated with 2 μM emetine for 1 h, PAR level was 67.7 ± 4.4 pg/10^6^ cells, which also was significantly higher than that of the control cells without emetine (27.4 ± 1.6 pg/10^6^ cells) (p < 0.01) (n = 2).

Because the presence of the PARG inhibitor might have caused this discrepancy, we further investigated how endogenous PAR level is influenced by various concentrations of the PARG inhibitor, PDD00017273, in asynchronously growing HeLa cells (Fig. 7B). PAR level in the control (no inhibitor) was 52.2 ± 26.7 pg/10^6^ cells. Strikingly, PAR level was higher in HeLa cells cultured for 30 min with the inhibitor (per 10^6^ cells): 0.3 μM, 910 ± 458 pg; 1 μM, 1,831 ± 918 pg (p < 0.05); 3 μM, 2,323 ± 1,245 pg (p < 0.01). With 3 μM PDD00017273, the increase was 40-fold over the control. The increased *in vivo* level of PAR was comparable to results obtained with the DNA-damaging agent, MNNG ( Fig. Supporting information Fig. S2).

### 3.10. Purified PARP1 has intrinsic activity and activated by histone H4 in vitro in the absence of DNA

**Fig. 8.**
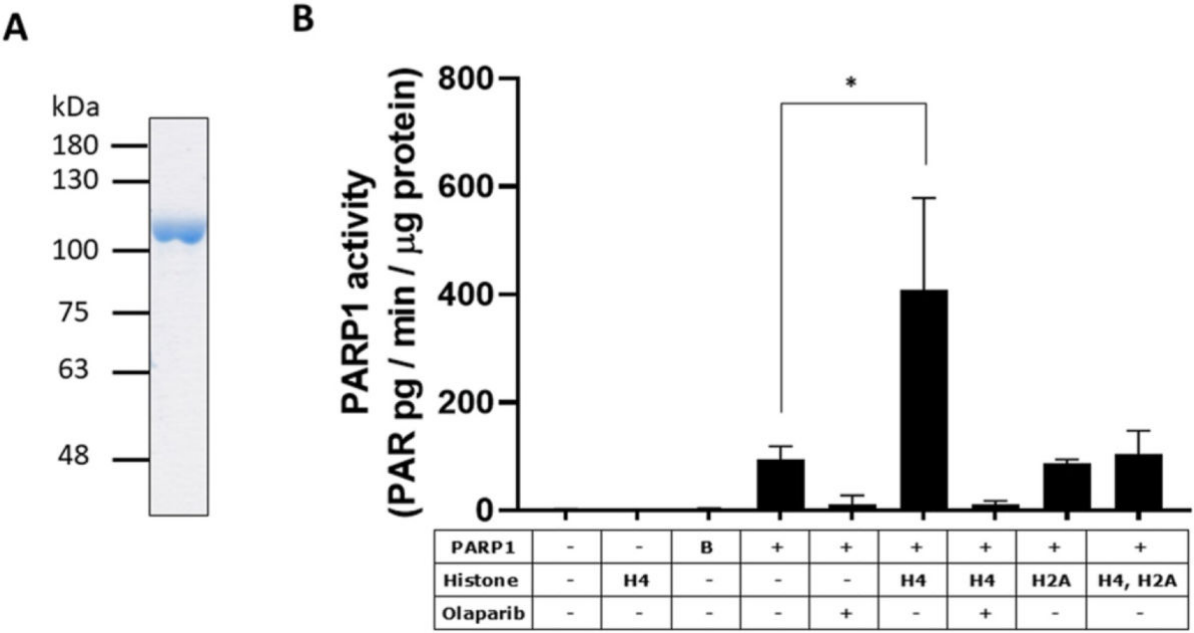
Intrinsic PARP1 activity in the absence of DNA and its activation by histone H4. (A) Purified human PARP1. PARP1 (2.0 μg) was separated via SDS-PAGE and stained with CBB. (B) Purified human PARP1 was incubated without or with human histones, and its activity was determined with ELISA. One unit of PARP1 activity is defined as 1 pg PAR formed/min/μg protein. n = 4. The significance was analyzed without or with histone H4 in the presence of PARP1 by two-tailed *t*-test. *p < 0.05.

Kim et al. reported that PARP1 activity was efficiently stimulated by nucleosomes under normal physiological condition and can regulate chromatin structure [61]. Subsequently, Pinnola et al. found that the nucleosomal core histone H4 in *Drosophila* chromatin stimulated PARP1 activity without DNA *in vitro* [23]. To test this possibility, it is crucial that PARP1 preparation is free from contaminating DNA. Therefore, we purified human PARP1 to confirm that it also can be activated by human histones (Fig. 8A).

Interestingly, the purified human PARP1 had measurable intrinsic activity in the absence of exogenous DNA or histones (Fig. 8B). Because exogenous DNA is supposedly essential for PARP1 activation *in vitro*, this low-level basal activity has not been analyzed in-depth in previous studies [20,62,63]. To determine whether this activity could be attributed to any contaminating DNA, we measured the activity of purified PARP1 at 100 nM in the absence or presence of exogenous nicked DNA of defined sequence. When 1 pg of immunodetectable PAR produced/min/μg protein is defined as 1 unit of PARP1 activity, 35 units of PARP activity was found in the absence of exogenous DNA. Units in the presence of nicked DNA were as follows: 0.1 nM DNA, 66 units; 1 nM DNA, 120 units; 10 nM DNA, 639 units; 100 nM DNA, 1,170 units. These results suggested that any contaminating DNA, if present, was equivalent to <1 nM of nicked DNA, implying that our purified PARP1 preparation was >99% free of DNA, with the assumption that nicked DNA binds PARP1 at a molar ratio of 1:1. Notably, the basal PARP1 activity increased by 300% upon addition of human histone H4 in the absence of exogenous DNA (p < 0.05) (Fig. 8B). Inclusion of both histone H4 and H2A resulted in a significant decrease in PARP1 activity, nearly canceling the increase brought about by H4 alone (Fig. 8B) (p < 0.05). These effects of human histones confirmed results reported by Pinnola et al. [23].

## 4. Discussion

Here, we found that the physiological level of PAR was quite low but quantifiable throughout HeLa cell cycle by eliminating various artifacts, and found that the PAR level of the HeLa cell cycle under normal condition, without using PARG inhibitor, peaks during S phase and is lowest in G1. To understand the physiological impact of PARylation on cell proliferation, a number of biochemical studies of PARP activity during the cell cycle have been performed using isolated nuclei, chromatin and nucleotide-permeabilized cells with conflicting conclusions [27,28,30, 32]. In this study, however, we clarified that the physiological PAR level in intact cells, throughout whole cell cycle is completely different from the reported PARP activity *in vitro*. These discrepancies could be primarily attributable to the facts that the PARP activity using isolated nuclear preparation, chromatin or nucleotides-permeabilized cells was influenced by possible DNA damage occurring during cell preparation, causing artificial activation of PARPs *in vitro* [31,34] and/or to the differences between intact cells and artificially modified cell components that lack low molecular weight metabolites leaking from the cells, which could influence *in vivo* enzyme activities [64]. Kidwell and Mage reported a small peak in PAR level during S phase in HeLa cells. However, their highest peak of PAR was in G2 when *in vitro* PARP activity of the isolated nuclear preparation was highest, and the smaller peak in S phase, when *in vitro* PARP activity was negligible [29]. Their highest PAR level in G2 was 200 ng/10^8^ HeLa cells, which corresponds to 2 ng (3.7 pmol)/10^6^ HeLa cells, was 80-fold higher than ours in G2/M (Fig. 3C). This difference might partly be attributable to the artifactual synthesis of PAR during lysis of large amounts of cells, 2.5–5 × 10^7^ cells, without using TCA to stop artificial PAR synthesis. The physiological PAR level of asynchronized HeLa cells determined with our ELISA is 0.04 pmol/10^6^ HeLa cells (Fig. 3C), which is consistent with the recent report by Martello et al., where TCA was used to fix cells, avoiding artifactual PAR synthesis in asynchronized HeLa cells and where PAR level was determined as 0.03 pmol/10^6^ cells using stable isotope dilution LC-MS/MS system [65].

The rapid turnover of PAR also implies the necessity of new PAR synthesis and its coupled degradation by PARG especially in S phase, which is supported by recent reports that PARG inhibitor alone is sufficient to induce a significant reduction in replication fork progression in the absence of genotoxic stress [19]. In addition, unperturbed DNA replication involves activation of a low-level inherent DNA damage response, including an activation of ATR-CHK1 [66]. The PAR could bind and activate Chk1 at stalled replication forks for S-phase checkpoint activation [67]. The rapid turnover of PAR contrasts with the relatively long half-life of NAD^+^, which is ~1 h in the HeLa line D98/AH2 [68]. Thus, the significant decrease of NAD^+^ level in S phase could be consistent with the rapid turnover of PAR chains (Fig. 5). In fact, the decrease of NAD^+^ level in S phase (4 h after release) was prevented by PARP inhibitors, particularly by olaparib (Fig. 5C and D). This finding suggests that about 20–25% of cellular NAD^+^ is used for PARylation in normal S phase cells. This notion is supported by the report that 3AB prevented the decrease of NAD^+^ level during S phase in FL cells [54]. The reason for the apparent increase of NAD^+^ level by olaparib in G1 phase (16 h after release from G1/S boundary) is not clear but it might be due to some remaining cell populations in G2/M phase (Fig. 2B), which showed higher NAD^+^ levels (Fig. 5A). Noteworthy, although 10 mM 3AB suppressed intracellular PAR level to 16% of the control level, the inhibition became less during the culture (Fig. 4). Cellular adaptive responses, including increased efflux of drugs [69], might be involved. Recently PARP inhibitors have been used as chemotherapeutic drugs for synthetic lethality, especially in those cancers with genetic defects of homologous recombination repair [13,14, 70]. Of note, long-term treatment of PARP inhibitor induced chemoresistance [71]. In addition, resistance to PARP inhibitor of *BRCA1/2* mutated cancer is due to the lowered activity of endogenous PARG [16]. The suitable assay method for measurement of cellular level of PAR should be also important for understanding the mechanism of drug resistance from the pharmacokinetic point of view.

We could confirm the effect of PARP inhibitors on suppression of cell proliferation and also S phase retardation with 3AB and olaparib. However, although 10 mM 3AB and 5 μM olaparib showed similar level of suppression of cell proliferation, we found some significant differences of flow cytometry profiles. This might be attributable to the fact that olaparib is a much more specific inhibitor of PARP1 and PARP2 [45] and, in addition, it can allosterically bind to PARP1 and PARP2 and to form strong PARP-DNA complexes [72–74], while 3AB is a general inhibitor of PARPs and inhibited the cell cycle, presumably not only in S phase but also in G2/M and G1, where other members of the PARP family, including tankyrase 1 and tankyrase 2, might also be inhibited [75]. The effect of clinically used PARP inhibitors on cell proliferation was reported to depend not only the *in vitro* effect of inhibition of PARP1, but also the effect of forming PARP-DNA complex *in vivo* [72–74]. It is interesting to measure the actual PAR levels in various cancer cells resistant to these PARP inhibitors [16].

Concerning DNA replication and PARylation, there have been studies reporting the association of PARP1 and DNA synthesome [76,77] and the binding of PARP1 to DNA polymerase α during S and G2 phases of the cell cycle [78]. However, although the involvement of PARylation on the change of chromatin structure and transcriptional regulation [61, 79] and the notable relationship between the relaxation of chromatin structure and PAR formation are available [80–83], little has been known about the involvement in PAR level during DNA replication *in vivo*, where a large change of chromatin structure including relaxation and condensation, should be occurring rapidly in a cell. The mechanism of the increase of PAR level in S phase is not due to the amount of PARP1, PARG, NAD^+^ or pERK2. Our data are consistent with Hanzlikova et al. in that PAR is mostly found in S phase. However, concerning the involvement of unligated Okazaki fragment as the increase of PAR in S phase [33], further work seems to be required to test the effect of emetine without or with PARG inhibitor in S versus G1 and to verify that emetine reduces DNA synthesis. We confirmed the findings by Pinnola et al. [23] that histone H4 stimulates PARP1 activity *in vitro* in the absence of DNA. While histones are synthesized all through the cell cycle of HeLa cells, their synthesis is greatly stimulated during S phase [84]. Thus, it is speculated that newly synthesized histones bind and activate PARP1, which will explain at least in part the increase of PAR in S phase. Further study to measure *in vivo* level of PAR is required to analyze the mechanism of regulation of PAR metabolism during the cell cycle.

## 5. Conclusion

First, we could quantify the basic *in vivo* level of PAR during whole cell cycle of HeLa cells without using PARG inhibitor and found that the *in vivo* PAR level is significantly higher in S phase than that in G1. Second, the half-life of PAR under non-stressed conditions is less than 40 s, consistent with the consumption of significant amount of NAD^+^ in S phase, which is rescued by PARP inhibitors. Third, PARP inhibitors delayed cell cycle in S phase. Fourth, the increase in PAR level during S phase is independent of the levels of PARP1, PARG, NAD^+^ and pERK2. Fifth, the purified PARP1 is activated by histone H4 in the absence of DNA.

## Research funds

This work was supported partly by Grants-in-Aid for Scientific Research (23590350) from the Japan Society for the Promotion of Science to M.M., and partly by Grants-in-Aid for Scientific Research from the Ministry of Education, Culture, Sports, Science and Technology-Japan (JP21H03547) to T.S. J. M. was supported by the Intramural Research Program, National Institutes of Health [NIH], National Heart, Lung, and Blood Institute [NHLBI]), U.S.A.

## Supplementary Material

Supplementary Material

## Abbreviations

3AB: 3-Aminobenzamide
BrdU: Bromodeoxyuridine
ELISA: Enzyme-linked immunosorbent assay
ERK: Extracellular signal-regulated kinase
pERK2: Phosphorylated extracellular signal-regulated kinase 2
HRP: Horseradish peroxidase
MEK: Mitogen-activated protein kinase kinase
MNNG: *N-*Methyl-*N*′-nitro-*N*-nitrosoguanidine
PAR: Poly(ADP-ribose)
PARG: Poly(ADP-ribose) glycohydrolase
PARP: Poly(ADP-ribose) polymerase
PARylation: PolyADP-ribosylation
PBS: Phosphate-buffered saline
TCA: Trichloroacetic acid
