# Supplementary material for: Physiological levels of poly(ADP-ribose) during the cell cycle regulate HeLa cell proliferation
Source: Exp Cell Res. Author manuscript; Available in PMC 2023 Aug 1. (PMC10009817; doi:10.1016/j.yexcr.2022.113163)
Supplement: Supplementary Material [file NIHMS1866687-supplement-Supplementary_Material.docx]

Appendix A. Supplementary data

The following are the Supplementary data to this article:
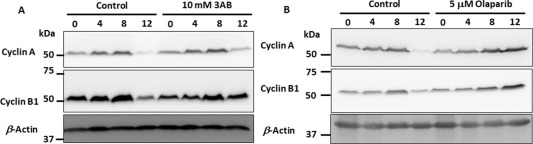


1. [Download : Download high-res image (110KB)](https://ars.els-cdn.com/content/image/1-s2.0-S0014482722001562-mmcfigs1_lrg.jpg)
2. [Download : Download full-size image](https://ars.els-cdn.com/content/image/1-s2.0-S0014482722001562-mmcfigs1.jpg)

Fig. S1. **3AB and olaparib delay cell-cycle progression.**

Levels of cyclin A and cyclin B1 in the presence of 3AB (A) or olaparib (B) at 0 h, 4 h, 8 h and 12 h after release from the G1/S boundary. Cells were subjected to Western blotting with anti-cyclin A and anti-cyclin B1 antibodies. β-Actin was used as the loading control. n=2.


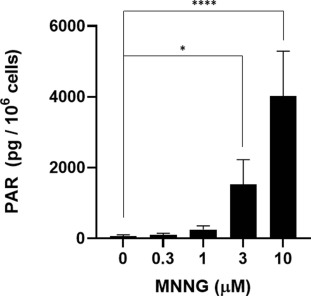


1. [Download : Download high-res image (81KB)](https://ars.els-cdn.com/content/image/1-s2.0-S0014482722001562-mmcfigs2_lrg.jpg)
2. [Download : Download full-size image](https://ars.els-cdn.com/content/image/1-s2.0-S0014482722001562-mmcfigs2.jpg)

Fig. S2. **The endogenous PAR levels without or with MNNG.**

Asynchronized HeLa cells were treated without or with the DNA-damaging agent, MNNG, for 30 min at 37°C, washed with PBS, immediately fixed with ice-cold 20% TCA, and processed for determination of PAR level by ELISA. HeLa cells were cultured in the medium containing the indicated concentration of MNNG with 0.01% DMSO. Control cells were cultured in the medium containing 0.01% DMSO (vehicle). The significance was analyzed by one-way ANOVA and Dunnett’s multicomparison test. n = 4. *p < 0.05, ****p < 0.0001.


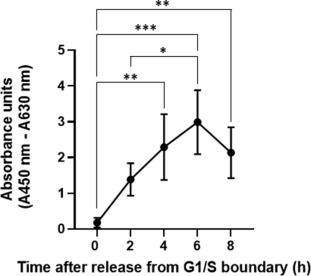


1. [Download : Download high-res image (92KB)](https://ars.els-cdn.com/content/image/1-s2.0-S0014482722001562-mmcfigs3_lrg.jpg)
2. [Download : Download full-size image](https://ars.els-cdn.com/content/image/1-s2.0-S0014482722001562-mmcfigs3.jpg)

Fig. S3. **Bromodeoxyuridine incorporation after release from the G1/S boundary.**

Bromodeoxyuridine (BrdU) was added to the cells for 15 min, and then DNA synthesis was assayed. The significance was analyzed by one-way ANOVA and Tukey’s multicomparison test. *p < 0.05, **p < 0.01, ***p < 0.001.


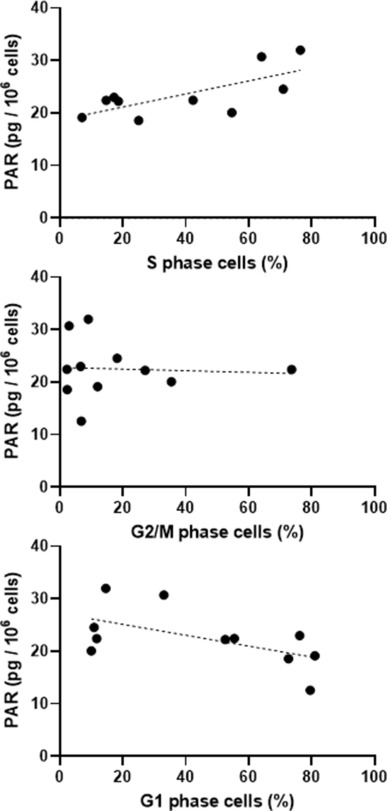


1. [Download : Download high-res image (209KB)](https://ars.els-cdn.com/content/image/1-s2.0-S0014482722001562-mmcfigs4_lrg.jpg)
2. [Download : Download full-size image](https://ars.els-cdn.com/content/image/1-s2.0-S0014482722001562-mmcfigs4.jpg)

Fig. S4. **Positive correlation between PAR levels and S phase.**

HeLa cells were synchronized with double-thymidine method and PAR levels were analyzed during cell cycle. The correlation between PAR levels and population of cells at S phase (A), G2/M phase (B) and G1 phase (C) was analyzed by the test of no correlation with the R statistical package. The r values were 0.72 (p=0.013), -0.06 (p=0.86) and -0.57 (p=0.066) for S, G2/M and G1 cells population, respectively.


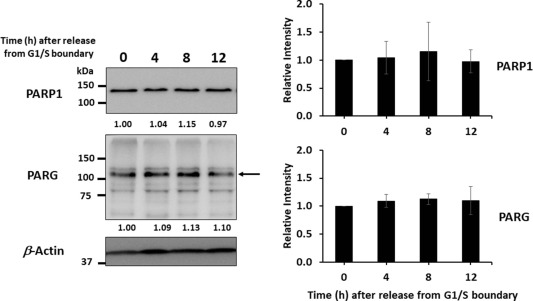


1. [Download : Download high-res image (222KB)](https://ars.els-cdn.com/content/image/1-s2.0-S0014482722001562-mmcfigs5_lrg.jpg)
2. [Download : Download full-size image](https://ars.els-cdn.com/content/image/1-s2.0-S0014482722001562-mmcfigs5.jpg)

Fig. S5. **PARP1 and PARG levels do not change during the cell cycle.**

**(**A) Levels of cellular PARP1 and PARG were determined with Western blotting using anti-PARP1 and anti-PARG Ig. β-Actin was used as a loading control. The arrow indicates PARG, and the other bands represent nonspecific bands or a spliced form of PARG. Data for PARP1 and PARG are representative of six and four independent experiments, respectively. (B) The relative intensities of PARP1 and PARG to β-actin were normalized to that at 0 h, after release. The significance was analyzed by one-way ANOVA and Tukey’s multicomparison test.
